# Semitransparent Organic Photovoltaic Devices: Interface/Bulk Properties and Stability Issues

**DOI:** 10.3390/nano14030269

**Published:** 2024-01-26

**Authors:** Barbara Paci, Flavia Righi Riva, Amanda Generosi, Marco Guaragno, Emanuela Mangiacapre, Sergio Brutti, Michael Wagner, Andreas Distler, Hans-Joachim Egelhaaf

**Affiliations:** 1 SpecX-Lab, Istituto di Struttura della Materia CNR, Via del Fosso del Cavaliere 100, 00133 Roma, Italy; 2 Dipartimento di Chimica, Università di Roma La Sapienza, P. Le Aldo Moro 2, 00185 Roma, Italy; 3 Helmholtz-Institute Erlangen-Nürnberg (HI-ERN), Forschungszentrum Jülich GmbH (FZJ), Immerwahrstraße 2, 91058 Erlangen, Germany; 4 Institute Materials for Electronics and Energy Technology (i-MEET), Department of Material Science, Faculty of Engineering, Friedrich-Alexander-Universität Erlangen-Nürnberg (FAU), Martensstraße 7, 91058 Erlangen, Germany

**Keywords:** in-situ characterization, structural properties, morphology, interfaces, OPVs, Ag nanowires

## Abstract

In the present work, an insight on the morpho/structural properties of semitransparent organic devices for buildings’ integrated photovoltaics is presented, and issues related to interface and bulk stability are addressed. The organic photovoltaic (OPV) cells under investigation are characterized by a blend of PM6:Y6 as a photo-active layer, a ZnO ETL (electron transporting layer), a HTL (hole transporting layer) of HTL-X and a transparent electrode composed by Ag nanowires (AgNWs). The devices’ active nanomaterials, processed as thin films, and their mutual nanoscale interfaces are investigated by a combination of in situ Energy Dispersive X-ray Reflectometry (EDXR) and ex situ Atomic Force Microscopy (AFM), X-ray Diffraction (XRD) and micro-Raman spectroscopy. In order to discriminate among diverse concomitant aging pathways potentially occurring upon working conditions, the effects of different stress factors were investigated: light and temperature. Evidence is gained of an essential structural stability, although an increased roughness at the ZnO/PM6:Y6 interface is deduced by EDXR measurements. On the contrary, an overall stability of the system subjected to thermal stress in the dark was observed, which is a clear indication of the photo-induced origin of the observed degradation phenomenon. Micro-Raman spectroscopy brings light on the origin of such effect, evidencing a photo-oxidation process of the active material in the device, using hygroscopic organic HTL, during continuous illumination in ambient moisture conditions. The process may be also triggered by a photocatalytic role of the ZnO layer. Therefore, an alternative configuration is proposed, where the hygroscopic HTL-X is replaced by the inorganic compound MoOx. The results show that such alternative configuration is stable under light stress (solar simulator), suggesting that the use of Molybdenum Oxide, limiting the photo-oxidation of the bulk PM6:Y6 active material, can prevent the cell from degradation.

## 1. Introduction

Semitransparent-solar cells (STCs) are a class of devices exploiting the benefits arising from the combination of photovoltaic (PV) properties and visible light transparency [1]. The technological relevance of STCs has been growing in recent years, mainly due to the opportunity of their integration in energy-sustainable buildings. The development of building-integrated photovoltaics (BIPV) is driven by current sustainability energy requirements aiming at reducing the dramatic impact deriving from the massive consumption of fossil fuels. Since decarbonization of energy systems requires a significant expansion of the PV areas, the integration of STCs as windows and skylights within buildings surfaces represents a big opportunity to greatly improve the energy sustainability in urban territories [2]. This aspect is extremely relevant in the perspective of the implementation of a CO_2_-neutral energy system, especially considering that cities are key-players in terms of energy consumption and climate impact [3]. Ideally, STCs should be characterized by high power conversion efficiency (PCE), while sustaining a high average visible transmission (AVT). Therefore, the utilization efficiency of photons in the ultraviolet (UV) and near infrared (NIR) components of the solar spectra should be maximized, while keeping an appropriate balance between light absorption and transmission in the visible region [4]. The opaque nature of crystalline Si-based solar cells has recently further motivated the interest in the realization of novel efficient transparent PV devices with promising innovative applications as BIPV systems: highly efficient STCs designs include perovskite [5,6], dye-sensitized [7,8] and organic-based systems [9,10]. Organic solar cells (OSCs), employing organic semiconductors with adjustable optical band gap, have a great potential as active materials in STCs as their absorption energies are strongly dependent on their molecular structure and can be easily tuned by proper selection of appropriate organic materials, in order to optimize the photon harvesting in the UV and NIR and the transmittance in the Vis. In addition to the band gap tunability, organic semiconductors are also characterized by high absorption coefficients, which limit the thickness of the organic material to about 100 nm for efficient light harvesting [4]. Typical designs for semitransparent organic solar cells (ST-OSCs) consist in multi-layers structures where the absorber is deposited as a thin film consisting in a bulk hetero-junction (BHJ) made of an interpenetrating continuous network of electron-donating (D) and electron-accepting (A) polymers/organic molecules blended together. In this configuration, the active layer is sandwiched between two conductive electrodes, where the charges generated at the BHJ after light absorption are collected. In order to facilitate efficient charge separation and transport, additional layers between the active material and the electrodes can be used, acting as electron transport layers (ETL) and hole transporting layer (HTL) [11]. Despite the performances of ST-OSCs in terms of efficiencies and stability being still not comparable with the standard Si technology, organic-based systems are extremely advantageous as they can be easily fabricated on a large scale by low-cost solution processes methods [12], even on flexible substrates [13,14]. Since the overall performances of ST-OSCs strongly depend on the properties of the transparent electrode and of the active layer, much effort has been recently dedicated to the attempt of realizing efficient ST-OSCs by a proper selection and engineering of the materials in the multilayer architecture. Many different transparent electrodes have been developed, including ultrathin metal Ag and Au electrodes and dielectric/metal/dielectric multilayer structures [15,16,17], transparent conductive oxides films [18,19], AgNWs networks [20,21,22,23], graphene [14,24], carbon nanotubes [25,26] and conducting polymers films [27,28,29]. Furthermore, since one of the main challenges in the realization of ST-OSCs is improving PCE while keeping high AVT, many studies focused on the investigation of materials able to selectively harvest NIR photons, to be employed as photo-active layers in ST-OSCs with a good balance between PCE and visible transparency. Among them, nonfullerene systems based on low band gap semiconducting polymers recently gained wide attention. In particular, derivatives of the Y series are considered promising acceptor materials in efficient devices due to their high carrier mobility and absorption in the NIR [30,31,32]. The advent of nonfullerene acceptors has boosted the performances of OSCs, with PCE reaching values up to 17% [33,34]. Furthermore, a considerable effort has been dedicated to the optimization of the efficiency and stability of the cells by proper materials and device designs, including efficient encapsulation strategies [35,36] and the implementation of tandem structures, with a consequent PCE gain to values as high as 19% [37]. Operating lifetimes of several thousand hours have been reported for accelerated aging tests in small area OSCs [38,39] and upscaling of highly efficient OSCs has been lately demonstrated, with PCE of 12.6% and 11.7% recorded for 26 and 204 cm^2^ module areas, respectively [40]. Despite the possibility of scaling up OSCs with optimized efficiency being essential in the perspective of their commercialization and big progress being made in making OSCs a valuable alternative to the traditional Si-based cells, stability still remains a big limiting issue for the applicability of this technology. Under real operation conditions, the possible factors concurring to the degradation of new PV devices’ generation may arise from an intrinsic instability (such as carrier recombination [41], phase separation [42] and residual lattice strain [43]) of the devices, as well as from extrinsic factors, such as mechanical stress [44], irradiation [45,46], heating [47,48] and exposure to O_2_ and H_2_O [49,50,51]. In particular, thermal degradation [52,53,54], photo-oxidation [55,56] and other photo-chemical/photo-physical processes [45,57,58] were found to dramatically affect the morphology of the active layer as well as of the carrier’s transport layers and the contact interfaces, with consequent detrimental effects on the overall performances of the device [59,60,61].

Among the nonfullerene systems suitable as active materials for ST-OSCs, BHJs based on PM6:Y6 blend have shown to be extremely promising potential in terms of performances and semitransparent properties, due to their high electron mobility, slow carrier recombination and broad and strong absorption for high photon harvesting in the NIR region [62,63,64]. PCEs of 15–16% and long operating lifetimes of thousands of hours have been reported for opaque devices using PM6:Y6 BHJ [65], with T80 lifetimes of over 4000 h [66], while for ST-OSCs with AVT, around 25% PCE only slightly decreases to 12–13% [67].

In this context, we present here a study of the structural and morphological stability of a ST-OSCs based on PM6:Y6 BHJ under light/thermal stress conditions. In the system under study, an AgNWs network was chosen as a transparent top electrode, in order to achieve the best compromise between high AVT and PCE for BIPV applications [68].

In situ EDXR [69,70] was used jointly with ex situ AFM, XRD and micro-Raman spectroscopy to evaluate the structural and morphological stability of the cell during exposure to solar simulator light. The combined use of these techniques, allowing to simultaneously investigate the morphology of the different device layers, is extremely valid in the study of multilayered OSCs. Indeed, the proposed method is able to detect bulk, surface and interface aging effects possibly occurring during operation. The effectiveness of such an approach lies in its ability to probe both the local and the overall morphology of the multi-layer, up to the angstrom resolution. Furthermore, the structural and compositional information needed to obtain insights on degradation phenomena possibly affecting the morphological and structural properties of the surfaces and interfaces upon illumination is gained. In particular, thanks to in situ EDXR, the evolution of the average morphological parameters is monitored in working conditions and related to the modifications locally affecting surface morphology (as detected ex situ by AFM) and to the composition/structure (as obtained by micro-Raman/XRD). In order to discriminate between concomitant aging pathways possibly occurring in stress conditions, the complete cell and the intermediate devices obtained at different steps of the cell fabrication were subjected to both light and temperature stress factors. The information on the structural, chemical and morphological degradation occurring at the surfaces and interfaces in the multi-layer is useful for the design of an alternative more stable device configuration.

## 2. Materials and Methods

The ST-OSCs under investigation exploit a PM6:Y6 BHJ ((Poly[(2,6-(4,8-bis(5-(2-ethylhexyl-3-fluoro)thiophen-2-yl)-benzo[1,2-b:4,5-b′]dithiophene))-alt-(5,5-(1′,3′-di-2-thienyl-5′,7′-bis(2-ethylhexyl)benzo[1′,2′-c:4′,5′-c′]dithiophene-4,8-dione)]): (2,2′-((2Z,2′Z)-((12,13-Bis(2ethylhexyl)-,9diundecyl12,13dihydro[1,2,5]thiadiazolo[3,4e]thieno[2″,3″:4′,5′]thieno[2′,3′:4,5]pyrrolo[3,2-g]thieno-[2′,3′:4,5]thieno[3,2-b]indole-2,10-diyl)bis(methanylylidene))-bis(5,6-difluoro-3-oxo-2,3-dihydro-1H-indene-2,1-diylidene))dimalononitrile)) as photo-active layer, ZnO and HTL-X (a poly(3,4-ethylenedioxythiophene) (PEDOT)-based ionomer), respectively, as ETL and HTL, and an AgNWs (silver nanowire) network as transparent top electrode.

Sample preparation: Prestructured glass/ITO substrates by Liaoning Yike Precision were cleaned in an isopropanol ultrasonic bath. Subsequently, zinc oxide (N-10 by Avantama) was doctorblade-coated onto the substrates and annealed at 200 °C for 30 min in air. The photo-active material PM6:Y6 (donor:acceptor ratio: 1:1.2, total solid content: 22 mg/mL in chloroform, materials purchased from Derthon OPV) was then blade-coated in air, before being annealed in the glovebox for 10 min at 110 °C. For semitransparent devices, HTL-X from RaynergyTek was blade-coated on top in air before being annealed in the glovebox for 3 min at 120 °C. These devices were finished by an in-air blade-coated AgNWs (Ink-Y by Cambrios) layer before being annealed again in the glovebox for 3 min at 120 °C. Laser-patterning of the AgNWs top-electrode defined the active cell area of 0.1 cm^2^. For the alternative configuration of the devices, 10 nm molybdenum oxide (MoOx) and 100 nm silver (Ag) were thermally evaporated subsequently without additional annealing or laser patterning step. The cell area was also 0.1 cm^2^ in this case.

Sample characterization:

Vis-NIR absorption of the samples was measured with a Shimadzu UV1800 spectrometer (Shimadzu, Kyoto, Japan), with an operating range from 190–1100 nm.

The current–voltage (I/V) curves of the solar cells were measured with a Keysight B2901A source measure unit and an LOT Quantum Design class AAA solar simulator, which provides 1000 W/cm^2^ of AM 1.5 G illumination.

EDXR measurements were carried out using an in-house patented energy dispersive X-ray reflectometer [71]. The experimental set-up exploits a nonsymmetric configuration to maximize the reflection of a poly-chromatic incident radiation produced by a W-anode in the energy range 10–50 keV and a detection system, consisting in an energy-sensitive solid-state, high-purity Ge-single crystal detector (ORTEC) kept at cryogenic temperatures via an electro-mechanical cooler. The focusing of the incident/reflected pathways was accomplished by W rectangular slits with horizontal and vertical apertures of 1000 μm and 40 μm, respectively. An Al (2 mm) filter was used to reduce the W-anode fluorescence lines. All the acquisitions were performed at fixed incident angles of 0.125° and 0.350°. For the in situ aging of the samples under light stress conditions, an integrated Solar Simulator AM 1.5 was jointly used while collecting the EDXR data in a N_2_-controlled atmosphere. In situ real-time EDXR measurements during thermal treatments were performed in air at 80 °C, using a specially designed heating sample holder.

XRD measurements were performed in Bragg–Brentano configuration using a Panalytical Empyrean X-ray diffractometer equipped with a PixCel 3D detector working in linear mode, a flat sample holder for thin films and a Cu-anode X-ray source (K-Alpha1 [Å] = 1.54060; K-Alpha2 [Å] = 1.54443). The XRD patterns were collected in the 5° < 2θ < 70° angular range (Gonio acquisition, Step Size [°2θ] = 0.0260, Scan Step Time [s] = 1145, Scan Type Continuous), setting the incident optical pathway by divergent slits of 0.2177°.

Micro-Raman measurements were performed in static mode by an InVia Renishaw micro-Raman apparatus, equipped with a 457 nm laser-25 mW and edge filters. Accumulations and scan times, as well as the laser power, were optimized for each sample, while a 2400 l/mm grating and a 100× microscope objective were used for all the measurements. The semitransparent devices and all intermediate samples were measured with the AgNW electrode or functional layers facing the incoming laser. The complete cell with evaporated Ag electrode was measured with the glass facing the incoming laser. AFM morphological characterizations were carried out by an in-house developed Atomic Force Microscope equipped with a 30 μm × 30 μm scanner. Several acquisitions representative of different portions of each sample were collected in noncontact mode by means of aluminum coated standard tapping AFM probes (Nanosensors, Neuchatel, Switzerland).

## 3. Results

**Figure 1:**
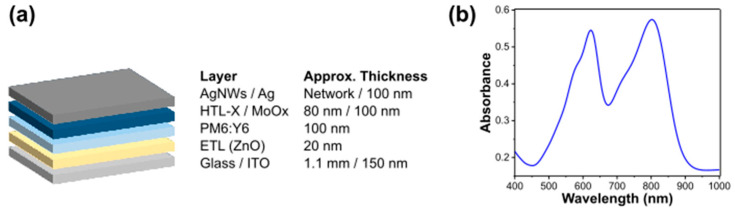
(**a**) Schematics of the solar cell stack and (**b**) Vis-NIR absorption spectrum of PM6:Y6 forming the BJH active material of the OPV device.

In this work, stability issues in OPV devices for BIPV applications are investigated, focusing on the relationship between the morphological/structural properties, interface and bulk stability and the photovoltaic performances of the devices. The architecture of the inverted solar cell is shown in Figure 1a. The semitransparent devices comprise an 80 nm HTL-X layer and an AgNWs network as the top electrode, while in the alternative configuration the devices have a 10 nm MoOx and a 100 nm silver top electrode (Ag). The absorption spectrum of the active layer (PM6:Y6) is shown in Figure 1b and features two main absorption bands, one centered at 620 nm (PM6 absorption) and one centered at 800 nm (Y6 absorption). The devices under study are characterized by current–voltage (I/V) measurements under illumination with “1 sun” (1000 W/m^2^ AM 1.5 G).

The photovoltaic key parameters of all cells are summarized in Table 1. The devices with AgNWs top electrode show a lower power conversion efficiency (PCE = J_sc_*V_oc_*FF) than the cells with evaporated silver top electrode. While the difference in open-circuit voltage (V_oc_) is rather small, the relative difference in fill factor (FF) is ~16%, which may be due to the higher sheet resistance of the AgNWs electrode compared to evaporated silver. However, the largest discrepancy (~32%, relatively) is found in the short-circuit current (J_sc_), which can be explained by the fact that the cells with AgNWs are semitransparent, as strictly required for BIPV applications, i.e., a significant amount of light is transmitted through the device and not back-reflected into the device as for the compact silver electrode. Consequently, less light can be converted into electric power, which leads to a lower J_sc_. It is worth mentioning that the PCE values reported in Table 1 for both devices are quite remarkable compared to the typical efficiencies of OSCs and matches well with the available literature on analogous systems [22,67].

**Figure 2:**
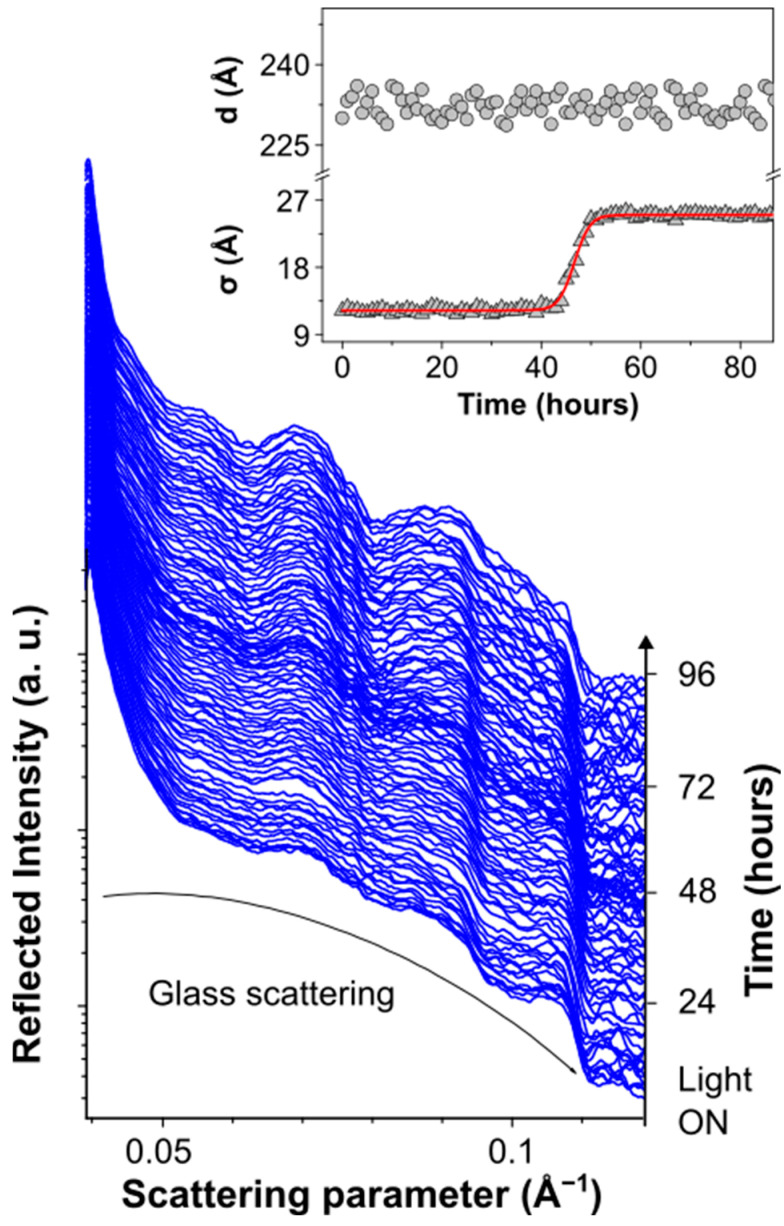
Time-resolved in situ EDXR measurements collected upon 96 h illumination on the semitransparent device. In the inset, the time evolution of the morphological parameters, as deduced by the Parratt fitting of the EDXR patterns. The sigmoidal fitting of the roughness evolution in shown as red line.

The structural and morphological stability of the multilayered OPV devices was studied under different stress conditions by a combination of in situ EDXR and ex situ XRD, AFM and Raman measurements. The synergistic use of these techniques allowed us to obtain insights on the main surface and interface effects possibly affecting the structural, morphological and chemical stability of the device layers upon illumination. In order to elucidate the role of each layer on the overall device stability, the materials in the complete cells were investigated as well as in the intermediate cells resulting from the different steps of the device fabrication. Figure 2 shows the time-resolved EDXR spectra of the glass/ITO/ZnO/PM6:Y6/HTL-X/AgNWs multilayer measured in situ during 96 h illumination under solar simulator. The EDXR patterns highlighted an increase in the roughness at the ZnO/PM6:P6/HTL-X combined interfaces with a temporal evolution following a sigmoidal trend between t = 40 h and t = 50 h. This effect may be related to the photo-oxidative degradation of the active layer. An analogous behavior has indeed been reported in the literature for several conjugated polymers [58,72,73,74] and attributed to modification of the chemical structure of the materials governed by lateral chain scission and cross-linking reactions, leading to a reduction in the π-conjugation with a consequent dramatic evolution of the film morphology and roughness.

**Figure 3:**
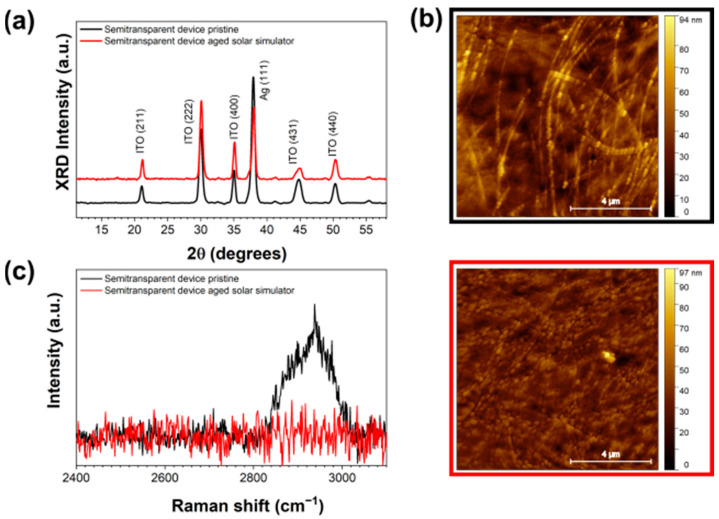
Ex situ characterization of the semitransparent device: (**a**) XRD and (**b**) micro-Raman measurements before (black line) and after (red line) 96 h under solar simulator illumination. (**c**) AFM images collected on the pristine sample (top) and after illumination (bottom).

To better understand the origin of the observed increase in roughness, XRD, micro-Raman and AFM measurements were carried out ex situ on the semitransparent cell, before and after prolonged exposure to solar simulator light. XRD diffractograms (see the Figure 3a) show a decrease in the intensity of the Ag (111) Bragg reflection after illumination, that is indicative of a loss in crystallinity of about 40%, related to the pristine AgNWs. Importantly, no sign of organic molecules’ crystallization is found, evidencing the enhanced structural stability of the PM6:Y6 BHJ with respect to previously studied blends [75,76]. In the case of the pristine sample, the AgNWs network is clearly visible, as deduced from the AFM measurements reported in Figure 3b, and the surface morphology is qualitatively comparable with the AFM and FE-SEM observations reported for AgNWs/PEDOT:PSS electrodes [77]. Furthermore, by comparing the AFM images collected on the surface before (Figure 3b, top) and after (Figure 3b, bottom) illumination, a reduction in the surface roughness is observed, as the AgNWs network results in being mostly embedded in the HTL after exposure to light. The partial collapse of the NWs in the HTL-X bulk is compatible with their apparent crystallinity loss deduced by XRD. Furthermore, from the micro-Raman measurements in Figure 3c, indication of chemical modifications of the active materials can be related to the drastic drop in intensity of the PM6:Y6 characteristic band assigned to the C-H vibration modes around 2950 cm^−1^ [78] after illumination. These results, together with the in situ time-resolved EDXR in Figure 2, suggest the occurrence of degradation phenomena affecting the stability of the BHJ/HTL interface.

**Figure 4:**
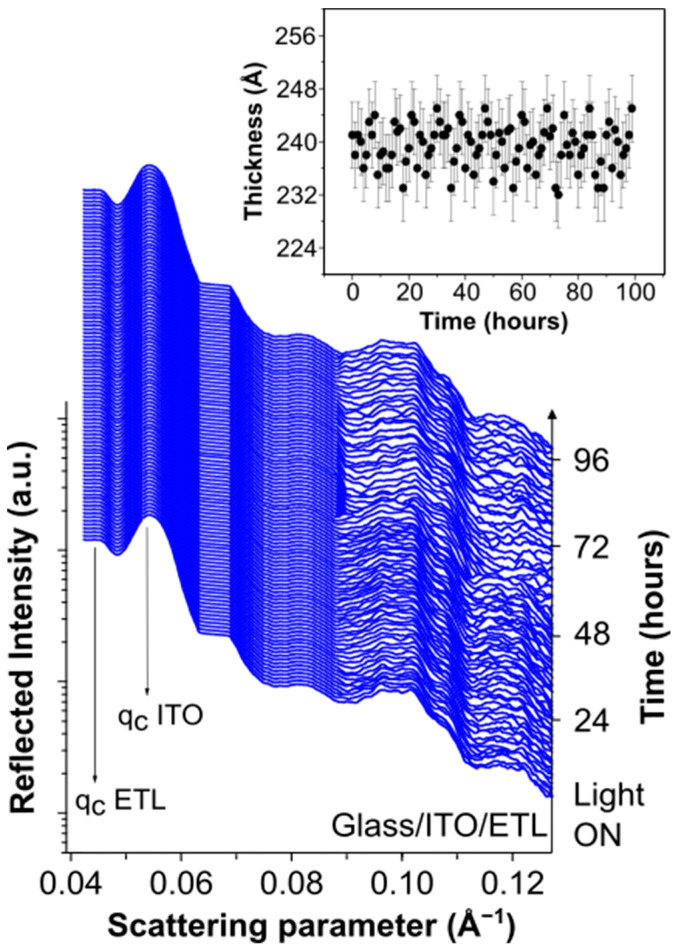
Time-resolved in situ EDXR measurements performed on glass/ITO/ZnO upon 96 h illumination.

To further support the above-mentioned hypothesis, time-resolved EDXR measurements during in situ illumination were performed on the exposed ETL. The results summarized in Figure 4 show that the intermediate device glass/ITO/ZnO is essentially stable during illumination as no clear changes occur in the time-resolved EDXR spectra, thus supporting the hypothesis that the enhanced roughness revealed by EDXR on the complete cell (see Figure 2) originated almost only from the PM6:Y6/HTL-X interface.

These results straightforwardly demonstrate the presence of degradation phenomena at the PM6:Y6/HTL-X interface affecting the morphological stability of the semitransparent cell during illumination. Based on the reported literature, we can speculate that such a detrimental effect is due to the photo-oxidation of the bulk PM6:Y6 and HTL-X components, which is triggered by free radical generation and accelerated in the presence of O_2_ [58,72,79]. However, since the OPV devices under illumination reach a temperature of about 80 °C, in order to rule out any thermal contribution to the aging pathway in working conditions, the semitransparent cells were characterized in situ during thermal treatment in the dark.

**Figure 5:**
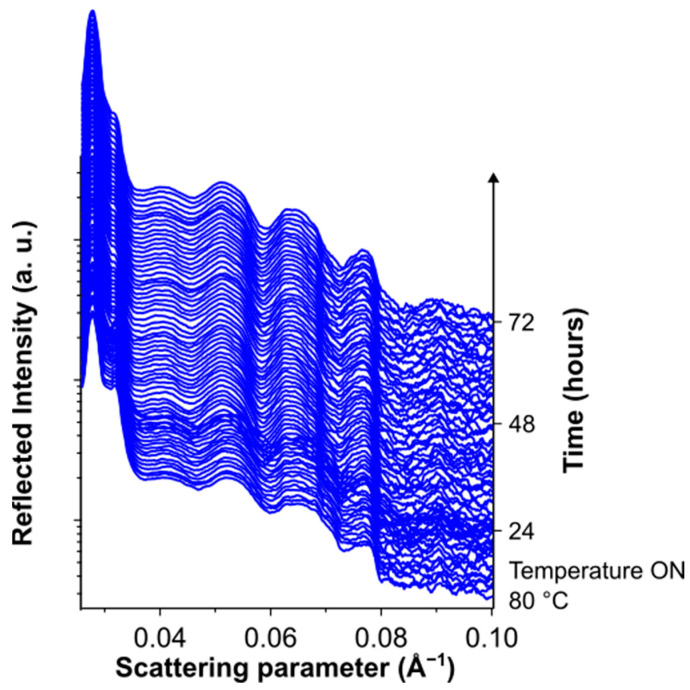
Time-resolved in situ EDXR patterns collected on the semitransparent device for 72 h under heating at 80 °C in the dark.

As shown in Figure 5, the time evolution of the EDXR spectra of the glass/ITO/ZnO/PM6:Y6/HTL-X/AgNWs device show a remarkable stability of the interfaces upon heating. The morphological parameters (thickness and roughness) of the surfaces and interfaces between the layers in the semitransparent cell, as derived from time-resolved in situ EDXR measurements, are almost constant during the thermal treatment. Only a slight roughness increase is revealed at the AgNWs exposed surface, thus supporting the hypothesis that the degradation of the cell during illumination (see Figure 2) is driven by photo-oxidative reactions triggered only by light absorption.

**Figure 6:**
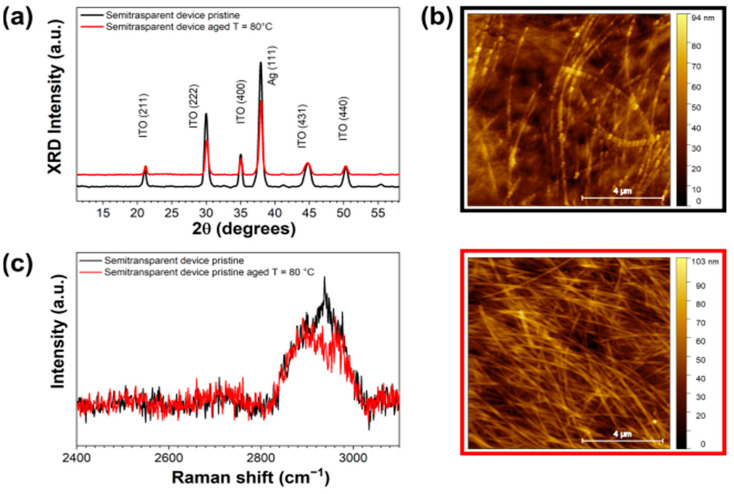
Ex situ characterization of the semitransparent device: (**a**) XRD and (**b**) micro-Raman measurements before (black line) and after (red line) 72 h heating at 80 °C in the dark. (**c**) AFM images collected on the pristine sample (left) and after thermal treatment (right).

Subsequently, the semitransparent complete device was characterized ex situ before and after prolonged heating, providing insights on the effects of temperature on the chemical, structural and morphological stability of the sample. Differently from what observed during exposure to the solar simulator, the thermal stress does not significantly affect AgNWs crystallinity, as shown by XRD in Figure 6a, nor the characteristic PM6:Y6 Raman band (see Figure 6c), thus indicating that the active layer is chemically stable upon heating. As shown in Figure 6b, only a slight roughness increase relative to the exposed surface is measured by AFM after aging, in agreement with the EDXR results in Figure 5. Such augmented roughness is mainly due to the AgNWs emerging from the HTL-X layer after heating, as shown in the bottom AFM micrograph of Figure 6c, where very long AgNWs appear clearly visible on top of the HTL surface. Such effect could be attributed to the hygroscopic character of the PFSA phase, causing the HTL micro-structure to be very sensitive to moisture and temperature variations [80]. The above observations suggest that the cell is essentially stable to the thermal stress factors, thus confirming the fully photo-induced origin of the degradation phenomena observed in stress conditions.

As reported by Tournebize et al. in [58] and by Manceau et al. in [72], photo-degradation of poly(2,7-carbazole) and poly(3-alkylthiophenes) derivatives is driven, respectively, by the scission of the C-N bonds and by the oxidation of the alkyl side-chains, and by the sulfur atom in the thiophenic rings. In the case of the system under investigation, the former reaction can be reasonably expected only for the P6 compound, while photo-oxidation of the lateral chains and of thiophene rings can occur for both PM6 and P6 as well as for HTL-X. Such reactions, which ultimately lead to a chemical and morphological degradation of the involved materials, are all triggered by the formation of free radicals activated by the absorption of light. Furthermore, environments containing large amounts of oxidative agents, such as O_2_ and H_2_O promote the occurrence of the above reactions. In order to overcome the limitations deriving from the direct exposure of the organic layers to the environmental atmosphere and moisture, we propose an alternative device configuration where the hygroscopic HTL-X layer and AgNWs network are replaced, respectively, by an inorganic HTL of MoO_x_ and a continuous thin film electrode of evaporated Ag. The use of an inorganic HTL has, indeed, been demonstrated to improve the efficiency and stability in OPV devices, overcoming the limitations associated to the high hygroscopicity and acidity of most commonly employed organic HTL, such as PEDOT-PSS [81,82].

**Figure 7:**
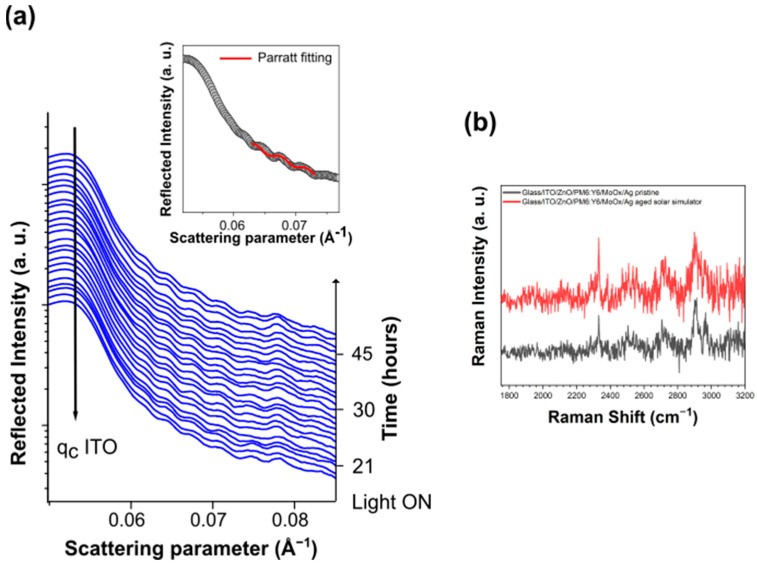
(**a**) Time-resolved in situ EDXR and (**b**) ex situ Raman spectra collected on the glass/ITO/ZnO/PM6:Y6/MoOx/Ag device upon prolonged exposure to solar simulator light.

Differently form the organic HTL cell, the in situ-time-resolved EDXR characterization and the ex situ micro-Raman measurements, reported in Figure 7a,b, revealed a complete stability of the chemical, morphological and structural properties of the surfaces and interfaces upon prolonged exposure of the MoOx-HTL device to the solar simulator light. Therefore, the above results confirm that the use of an inorganic MoOx-HTL and the replacement of AgNWs with a thin Ag film, acting as physical barrier against air and moisture, can effectively improve stability of ST-OSCs upon prolonged illumination.

## 4. Conclusions

We report here on the experimental study of the chemical, structural and morphological properties of ST-OSCs based on a PM6:Y6 BHJ active layer and an AgNWs transparent electrode for transparent photovoltaics. Thanks to the synergic use of in situ EDXR and ex situ AFM, XRD and micro-Raman spectroscopy, the morphological, structural and chemical stability of glass/ITO/ZnO/PM6:Y6/HTL-X/AgNWs semitransparent PV devices was studied, under different stress factors. XRD analysis evidenced an essential structural stability of the device, while AFM, EDXR and micro-Raman detected chemical/morphological degradation phenomena of the nanomaterials, processed as thin films, and of their mutual interfaces in the multilayered system. In particular, the study of the complete semitransparent cell and of the intermediate steps of the device, subjected to light and temperature stress factors, allowed to identify the occurrence of photo-degradation phenomena, mainly affecting the bulk properties of the active layer and the BHJ/HTL interface. These reactions, which ultimately may lead to a chemical and morphological degradation of the involved materials, occur through photo-oxidation of the lateral chains (and of thiophene) of PM6, Y6 and HTL-X, are triggered by the formation of free radicals and accelerated in presence of O_2_. Such effect may be further activated by a catalytic role of the ZnO layer. Therefore, in order to minimize the effect on the cell of oxidative agents commonly found in environmental conditions, the organic hygroscopic HTL was replaced by a MoOx layer. Importantly, no evidence of degradation was found at the BHJ/HTL interface from the in situ and ex situ characterizations of such device, suggesting that the use of an inorganic MoO_x_ HTL, limiting the photo-oxidation of the bulk PM6:Y6 active material, can increase the overall stability in stress conditions, preventing the cell from degradation.

Importantly, the approach proposed in the present work, for the in situ monitoring of nanomaterials and interfaces, is of general interest and can be extended to the study of different kinds of nanomaterial-thin films-based systems, as valuable strategy to develop efficient and stable devices.

## Figures and Tables

**Table 1:** Photovoltaic parameters (short-circuit current (J_sc_), open-circuit voltage (V_oc_), fill factor (FF) and power conversion efficiency (PCE)) of PM6:Y6-based solar cells with compact Ag top electrode and semitransparent AgNWs. The shown values are mean values and standard deviations of 12 individual cells per variation.

	J_sc_ [mA/cm^2^]	V_oc_ [V]	FF [%]	PCE [%]
Ag	22.8 ± 2.1	0.827 ± 0.002	65.7 ± 1.1	12.4 ± 1.0
AgNWs	15.4 ± 1.5	0.792 ± 0.006	55.2 ± 1.4	6.7 ± 1.4

## Data Availability

The data supporting the findings of this study are available from the corresponding author upon reasonable request.
